# The Hospital. Nursing Section

**Published:** 1904-05-21

**Authors:** 


### The Hospital.

### Nursing Section.

Contributions for this Section of "The Hospital" should be addressed to the Editor, "The Hospital" Nursing Section, 28 & 29 Southampton Street, Strand, London, W.C.

No. 921.—Vol. XXXVI. *SATURDAY, MAY* 21, 1904.

### Notes on News from the Nursing World.

### MISS FLORENCE NIGHTINGALE.

The King has been pleased to sanction the appointment of Miss Florence Nightingale as Lady of Grace of the Order of St. John of Jerusalem in England. On Thursday last week Miss Nightingale celebrated the eighty-fourth anniversary of her birth. She received at her residence in South Street, Park Lane, a large number of congratulatory telegrams and messages, and there were even more afternoon callers than usual. Miss Nightingale, who continues to enjoy fairly good health and to take an unabated interest in works of charity, was engaged with her private secretary during the morning, and the celebration of her birthday was of the quietest description.

### CLARA LOWE.

The death of Miss Clara M. S. Lowe last week has removed from among us a devoted woman, who spent a considerable part of her long life in nursing the sick in various parts of the world. The daughter of Sir Hudson Lowe and the niece of an officer who was killed at Waterloo, she was born at St. Helena in 1818 and was present at the Coronation Ball of Queen Victoria. About 1860 she began nursing work in the East End of London and was the first woman of gentle birth to go and live among the very poor. She nursed the sick with assiduity in the cholera visitation of 1868. Again, in the Franco-Prussian war she helped to tend the sick and wounded. Subsequently, in 1871, she went to Canada to assist Miss Macpherson's emigration scheme, and her experiences included the nursing of small-pox patients and taking charge of their hospital. Then she went to India to assist Miss Reade, of the South Arcot Mission, and returning a few years later to England, resumed her rescue work in the East End. Miss Lowe was not a trained nurse, but she had the nursing instinct largely developed, and in days when trained nursing was almost unknown her ministrations to the sick were greatly valued, not only because they were voluntary, but also because of her marked capacity. Until within a very short time of her death she was in full possession of her faculties.

### PLAGUE NURSING IN JOHANNESBURG.

With regard to the death of Miss Blake, at Johannesburg, from plague, which was mentioned in our columns a fortnight ago, we learn that she had passed unscathed through similar epidemics in Cape Colony. She contracted the disease and died about ten days after taking up her duties in the Plague Hospital. The other two nurses from Johannesburg Hospital—who were selected from a great number of volunteers—have so far escaped, and it speaks well for the arrangements of the Plague Hospital and for the nursing that the mortality has only been 73 out of 108 cases. The nursing of plague cases is arduous, because the strictest attention and the quickest observation are necessary. Collapse is common in any stage of the illness, and must at once be combated by stimulants and hypodermics of strychnine. Very large quantities of both strychnine and alcohol can be administered in plague. It is exceedingly difficult to diagnose pneumonic plague in its early stages, and nearly all the cases in Johannesburg have been pneumonic, not bubonic—the reverse of experience in India. A lady doctor has been engaged by the Board of the Johannesburg Hospital to examine all patients before they are admitted, so as to minimise the risk of the residents being brought in contact with persons suffering from the disease.

### THE LATE SECRETARY OF THE HOSPITAL FOR SICK CHILDREN.

Although the nature of the late Mr. Adrian Hope's official duties did not bring him into much personal contact with the nurses at the Hospital for Sick Children, they have abundant evidence that his interest in their comfort and happiness was very real and keen. It may not be generally known that it is to Mr. Hope's foresight, and his energy in raising the necessary funds, that the nurses owe the beautiful Home which was acquired and fitted up for their occupation in 1899 at a cost of some £34,000. Having once become convinced of the necessity for improving the nurses' accommodation, Mr. Hope did not rest until the Home was an accomplished fact, and the subsequent benefit to the health of the staff has proved that the outlay was a necessary one. The acquisition of these premises, formerly the Convent of St. John and St. Elizabeth, conferred upon the nurses another boon, namely, the garden, which is in constant use both by nurses and their little patients in fine weather. To the matron, Miss Gertrude Payne, and her office staff, Mr. Hope's death is a great loss; while his visits to the wards, when showing them to subscribers and other friends, were always pleasant to the nurses, to whom, as to everyone in the hospital, he was invariably kind and courteous. The matron, sisters, and nurses, besides being present at the memorial service in the Hospital Chapel on Saturday, sent a beautiful wreath of white lilies and lilies of the valley to Hopton, in Suffolk, where Mr. Hope is buried.

### STATE REGISTRATION AND PARLIAMENT.

The supporters of State registration are not very happily represented in the House of Commons. The other day, Dr. Thompson asked the President of the Local Government Board if he would consent to appoint a small Select Committee to consider the advisability of the State registration of nurses. Mr. Long, of course, had to explain that the subject of nursing generally is not within the province of his department, which is only concerned with it as respects nursing in Poor-law institutions. At the annual general meeting of the Society for the State Registration of Trained Nurses on Friday the subject was referred to and it was declared desirable, in the public interest, that a Select Committee should be appointed to inquire into the whole nursing question. The proper person to approach in Parliament is the Prime Minister.

### THE REPORT OF THE DEPARTMENTAL COMMITTEE.

On Monday the question of the action of the Local Government Board in reference to the report of the Departmental Committee upon the nursing of the sick poor in workhouses, came again before the House of Commons. Sir Harry Samuel asked the President of the Board whether it had been decided to issue a general order consequent upon the recommendation contained in the report; and if so, when such order would be issued. Sir Harry also inquired for what Parliamentary period before such issue the order would be laid upon the table of the House of Commons for its consideration. Mr. Long replied that his department was still engaged in considering the order, and that it would not have to lie upon the table before being issued.

### DR. BOXALL AND THE MIDWIFE'S AGE.

With reference to our remarks last week under the heading of "The Midwife's Age," Dr. Boxall informs us that the age limit referred to by him at the meeting of the Rural Midwives' Association was purely that for examination, and not for the practising age of the midwife. He adds that the age for examination was formerly 21 to 40, and has been only recently increased to 45 as an experiment. This, he points out, means that a qualified midwife can practise as long as she is physically able to do so. But we adhere to the opinion that 21 is too early an age for her to be permitted to qualify, much less to practise.

### UNIVERSITY COLLEGE HOSPITAL LADIES' ASSOCIATION.

The fourth general meeting of the University College Hospital Ladies' Association was held in the board-room of the hospital on Monday, Katharine Duchess of Westminster, presiding. The association now consists of 386 members, and has five branches, whilst a new one is about to be inaugurated at Wimbledon. The number of garments contributed for the use of destitute patients upon their convalescence or discharge from hospital was 1,464, 265 being made at a London working party. The association also contributes £100 a year for maintaining a medical bed called "The University College Hospital Ladies' Association Bed"; £80 of this had been collected by the Duchess of Devonshire. It is hoped later on to maintain a surgical bed in addition. The matron, at the close of the proceedings, thanked the members, on behalf of the sisters and herself, for their most welcome help. When they visited the wards they would see that nearly all the patients were wearing red flannel jackets provided by the association. Gifts of flowers, fruit, toys, books and convalescent home letters had been also much needed and appreciated. Ladies wishing to join the association should apply to Mrs. Bradford, 8 Manchester Square, W.

### THE CENTRAL MIDWIVES BOARD DIVIDED.

At the meeting of the Central Midwives Board, which was held on April 18, and adjourned to May 11, Dr. Champneys was re-elected chairman, and Mr. J. H. Johnstone, M.P., hon. treasurer for the ensuing year. The Board resolved to reconsider its rules so as to allow pupil midwives trained in the Rotunda Hospital, Dublin, the Royal Academy of Medicine in Ireland, and the Incorporated Belfast Maternity Hospital, to be placed on the same footing as candidates for examination producing the certificates prescribed by Forms III. and IV. in the schedule to the rules of the Board. But a resolution to the effect that, in lieu of the certificates of personal attendance upon 20 cases the Board should, if they think fit, accept the certificates of the master or senior medical officer of a hospital or institution where midwives are trained that the candidate has attended the course of training prescribed for pupil midwives for the period, and in accordance with the regulations in force in such hospitals or institutions, was lost by five votes to three, and the further consideration of the matter adjourned to May 26. The names of 1,037 women were ordered for entry on the roll, which now consists of 4,452 names. It was resolved to appoint an inspector to visit and report on institutions applying for recognition of certificates or approved as training schools, in cases where it appears advisable to the Board.

### LADY DUDLEY'S DISTRICT NURSES.

The first annual report in connection with Lady Dudley's scheme for the establishment of district nurses in the poorest parts of Ireland, covering the 12 months ended on the 23rd of last month, has been issued. The population of the agricultural districts in which eight out of the nine nurses have been started is about 23,000, and the districts are among the most remote in Mayo, Galway, Roscommon, Kerry, and Donegal. All of the nurses have been fully trained, either at St. Lawrence's or St. Patrick's Home, Dublin, according to their religion, and all are enrolled as members of Queen Victoria's Jubilee Institute. The annual cost of each nurse varies from £90 to £112, according to the charge made for lodgings. Each nurse is provided with either a rough pony and cart or a bicycle, and medical stores. The report does not include the accounts for the first working year's expenditure, as they are not yet complete, but it contains testimony from all sorts of people as to the value of the movement. Of the latter, that of the Rev. J. Browne, of Glengariff, seems to express the universal feeling. Mr. Browne says: "The visit of the nurse is hailed with delight, and her influence for good is not confined to the homes of the sick—her example s educating and refining the whole district."

### THE CORONER AND THE MATRON.

At an inquest held last week on the body of a labourer named Rowe, Dr. Taylor, the coroner for

Mid-Surrey, used rather strong language respecting the matron of Kingston Union Infirmary. Rowe was removed from his home at Wimbledon to Kingston Infirmary in a cab. He was suffering from pleurisy and pneumonia, and died two hours and a quarter after admission. The matron reprimanded the porter for allowing the man to walk from the cab to the ward, and afterwards made a complaint to the coroner's officer, in consequence of which an inquest was held. It was then stated that the medical officer and the steward were present when Rowe was admitted and that he walked to the ward with their approval; whereupon the coroner said that the matron had been "guilty of unwarrantable interference." Seeing, however, that she was clearly prompted by the kindliest motives, and that the death of the patient in two hours and a quarter proved his alarming condition on his admission, we do not think that there was any necessity for Dr. Taylor to employ a phrase which suggests a heinous offence rather than an indiscretion.

### THE MATRON OF THE NEW MATERNITY HOSPITAL, JOHANNESBURG.

We understand that Sister A. K. Statham, of the Army Nursing Service Reserve, who nursed soldiers in No. 6 General Hospital in the Transvaal all through the Boer War, and who nearly lost her life from enteric fever, has just been appointed matron of the new Maternity Hospital at Johannesburg. In May 1902 she was invalided home, and she has been the recipient of two war medals. Miss Statham, who only returned to South Africa in January last, was trained at Sussex County Hospital, Brighton, and has enjoyed a long and varied experience; among other equipments for her office she possesses a first-class diploma in housekeeping at the Edinburgh School of Cookery. She holds the certificate of the London Obstetrical Society.

### EAST END MOTHERS' HOME.

At the annual meeting of the East End Mothers' Home last week, presided over by Dr. Owen Lancaster, there was a large attendance of friends of the charity. In the report, of which Mrs. Joceline Bagot moved the adoption, it is stated that 344 poor women were received in the year as in-patients at the home, and that 422 had been attended in their own homes. In only eight cases—four in and four out—had the infants died. Mrs Bagot complimented the committee on the low average cost of each inmate's treatment, and dealt with the great value of care and good food to the after-health of the poor working mothers. The other speakers included the Hon. G. J. Goschen, M.P., who said that the home was non-sectarian; Dr. May Thorne, who dwelt on the valuable precepts of cleanliness, order, and quiet which the women acquired in the wards; and Mrs. Quintin Hogg, who enlarged upon its claims to personal sympathy and support.

### DEATH OF NURSE DYNE AT KING'S COLLEGE HOSPITAL.

The news of the death of Nurse Emily Mary Dyne, which took place at King's College Hospital last week, came as a shock to her large circle of friends. Miss Dyne had been nursing a case in Malta, and returned to England last Friday week. It was known that she had been far from well, and a lady whom she had nursed, and who was very devoted to her, had arranged for her to be received at King's College Hospital, where she was nursed through an illness some nine years ago. Directly after arriving at her lodgings in Bayswater, she was at once conveyed to the hospital, where she died on the following Wednesday. A tall, muscular, finely-built woman, Miss Dyne was devoted to her patients and to children, and of a most sympathetic disposition. She began her nursing career as a probationer at King's College Hospital, in 1877, in the days when the nursing was in the hands of the sisters of St. John's House. In 1883 she was staff nurse, and her L.O.S. certificate was taken in April 1884. Since July 1883, she appears to have been working as midwife and monthly nurse, her active service being continued almost up to the day of her death. Nurse Dyne's thoughtfulness for others is evinced by the fact that all the little gifts she brought home from Malta and Sicily, which she visited, were neatly labelled and addressed, and taken by her to the hospital. She was, however, too weak to deliver them to her friends, as she had planned. Nurse Dyne was buried at Oakley on Saturday.

### OUTRAGEOUS ASSAULT ON A CHARGE NURSE.

A charge nurse in Newcastle Workhouse Infirmary had an unpleasant experience the other day. One of the pauper inmates of the imbecile ward, a woman of 26, kicked the nurse on the legs and threw two basins of soup at her, breaking the basins over her back. The assault was in fact so serious that the case was brought before the Newcastle Police Court, and the offender was sent by the Bench to gaol for a month. It was suggested that she might be insane, but Dr. Cheese, the medical officer at the workhouse infirmary, stated that he could not certify her as a lunatic, though she had a very ungovernable temper. Her defence was that the nurse pulled her collar.

### QUEEN ALEXANDRA'S MILITARY NURSING SERVICE.

We are informed that Miss A. M. Pagan has been appointed staff nurse in Queen Alexandra's Imperial Military Nursing Service, and has been posted to the Royal Herbert Hospital, Woolwich. The following changes of station have been made: Sister M. G. Hill, R.R.C., has gone from Mooi River, Natal, to Pretoria; Sister L. M. Todd, from Pretoria to Mooi River, Natal; and Sister S. L. Wilshaw, R.R.C., from Devonport to Egypt.

### SHORT ITEMS.

We are informed that the late Sir Henry M. Stanley was nursed throughout his last illness by two male nurses who received their training at the National Hospital for the Paralysed and Epileptic. —Mrs. Clara Barton has resigned the presidency of the American Red Cross Society, and Mrs. John A. Logan is to be her successor.—Miss Keith has succeeded Miss Oldham as matron of the Hospital for Epilepsy and Paralysis.

### The Nursing Outlook.

"From magnanimity, all fear above;
From nobler recompense, above applause,
Which owes to man's short outlook all its charm."

### THE JAPANESE NURSE.

The daily papers give us occasional pretty glimpses of the serious little Japanese nurses, with their red crosses, giving tender and trained service to the wounded in the present war. We hear of them nursing the Russians with care equal to that expended on their own countrymen, but we hear no word of the Russian nurses. The fact is that in Japan trained nursing "caught on" as it did in England; whereas in Russia, as in France, nursing is in no way popular or fashionable, and enthusiasm in its cause cannot be raised.

It is 17 years since the first training school for nurses was organised in Japan in connection with the Empress's hospital, and with the support of the Government. Officials visited various countries beforehand and studied different methods, and finally invited Miss Linda Richards of Massachusetts to become superintendent of nurses at Kyoto, and to take with her as many American nurses as she thought desirable at the start. We wish the honour of organising trained nursing in Japan had fallen to England; however, we sent Alice Fisher to America years ago to start training schools there, so at second-hand the glory is still ours. And at Seoul, in Korea, there is a hospital started and worked by English Kilburn sisters, where Miss Robinson, of Dublin, is matron, and it will be interesting to hear in due course their experiences during this war. This must be the hospital to which the President of the Red Cross referred in his letter to us printed in our issue of May 7.

One of the main objects of the training schools in Japan was to found a new occupation for young women of position who needed to earn their living: therefore the Empress became patroness, and the first probationers were selected from the educated classes. Before these first pupils had completed their course they were all engaged to fill different posts at salaries we might perhaps scorn, but which represented excellent pay in Japan. Soon an opportunity came of testing the worth of the training given. In the Japan-China war of 1894–5 a call for nurses was made and the parent training school at once sent a matron and staff. It was a new thought for Japanese women to nurse men other than their own relations, especially under such conditions, but directly the trial came they responded promptly, and the results of their work were excellent. After this the pay of trained nurses went up considerably, and training schools spread all over the country. The Red Cross Society of Japan also reorganised on its nursing side and formed a training school, and the result was that in the North China troubles in 1900 the nurses served on the hospital ships as well as at the base hospitals. No wonder then that when Dr. McGee, on behalf of the "Spanish-American War Nurses" wrote last October to the Japanese minister at Washington offering to send a party of nurses to nurse the Japanese wounded in case of need, the reply was that the Minister was "deeply impressed" by the offer, and would pass it on to the proper authorities. In due course an acceptance came, and ten nurses from New York, Philadelphia, and other training schools were chosen. They have started, as volunteers without salary, to give such help as they can. Dr. McGee herself goes with them, to supervise their work, but she will not practise professionally on the wounded.

Miss Linda Richards says: "These nurses will be found in touch with every advance movement which is in any way connected with their profession. They reach out helping hands in every direction. Their services are sought and fully appreciated and their knowledge respected. They are valued members of society wherever they are to be found. They are ever on the alert, and though quiet and very modest, make steady and sure progress." In such ideal hands one can safely leave the Japanese and Russian wounded, especially after the late interesting account of the President of the Red Cross Society of Japan.

But we cannot resist quoting a most charming description of a Japanese nurse given by Mrs. Archibald Little, whose graphic pen gives the human interest which is so often lacking in mere reports. She says: "Her figure, the shape of a very soft feather pillow, was draped in a tight-fitting white apron with a large bib, and she was kept inside her buttonless and stringless clothes by a cruelly tight and wide leather belt. Into this belt, holding her breath for a long time, she could, with an effort, push her fat silver watch, her clinical thermometer, her carefully folded pocket handkerchief, and the relentless little register in which she noted down all the sins of my pulse and temperature. Oh! what a nurse she was! So gentle, so smiling, so very delightfully sorry for one! It was quite worth being ill to revel in such seas of sympathy! She had the real nurse's gift for feeling the time and waking at the right hour; and for eight days and nights I think she never failed to come to my bedside every two hours to replenish the ice-bags in which I lay." Mrs. Little goes on to describe her misery when for two days she had to put up with an untrained nurse: and indeed it is a grand thing to think now that in almost every civilised country, one can in time of sickness command a skilled woman attendant to care for one, even as though one were at home.

### Lectures Upon the Nursing of Mental Diseases.

By Robert Jones, M.D.Lond., B.S., F.R.C.S.Eng., M.R.C.P.Lond., Resident Physician and Superintendent of the London County Asylum, Claybury.

LECTURE XI. (*Concluded from page 85*).

In cases of (*f*) *epilepsy with insanity*, a form different from any hitherto considered, the patients are exceedingly querulous and irritable. They are, however, often peculiarly sympathetic with each other, and they may be seen in asylums in the gardens and grounds in groups, reading, talking, and associating together, and they are often devoutly religious, whereas those suffering from other varieties of insanity are each solitary and independent; unlike epileptics the ordinary insane have little or nothing in common. The prevailing feature of ordinary insanity is egotism or selfishness, which for the nurse is protective, as the patients might otherwise combine to overpower her, take her keys, and reverse the order of authority. In the case of epileptic insanity such is not the case, for they not infrequently combine to form antagonistic associations and cabals—which must be looked for and guarded against. Epileptic patients are vindictive, but there is a weakmindedness as the result of fits which not infrequently turns "king's evidence" and betrays the intriguer. Epileptic patients are more dangerous just before or immediately after a fit than at other times, but usually they are amenable to reason and can be tactfully managed without trouble between the epileptic seizures. The regulation of the bowels is a matter of great importance in the case of the insane, and especially in the case of the epileptic, for constipation favours the fits, and patients get to know this and to be relieved may ask for medicine when they need it. The "cry" of the epileptic precedes the fit, or rather is the first stage of it, and the nurse may often save a patient from falling if she can lay her on the floor or the ground when she hears the cry. All clothing round the neck should be at once released, a pillow placed under the head, and, when the paroxysm is over, the patient carried indoors or taken to a quiet retreat, as rest is needed for some time after the fit. One often hears of special instructions being issued to the nurses to prevent the biting of the tongue. I do not consider that any such precautions are necessary, as no precautions the nurse can take will be of any avail, and it is not a frequent or serious accident. She may loosen the teeth and hurt the patient by trying to give doubtful relief. For the rest of the day after a fit the patient is probably irritable and is best kept as much apart from sources of worry and anxiety as can be managed. All epileptic patients need special supervision by night, for they may sleep on their faces or sides, and may thus die from suffocation in a fit.

In the treatment of cases of (*g*) *imbecility or idiocy* the mental nurse may have some educational work to do, more especially in regard to habits of cleanliness, self help in dress and tidiness at table and in regard to clearness of articulation and speech. The term idiocy is used in regard to the more helpless class of imbeciles, the lower grade who are incapable of speech and who have no ability to dress, undress, wash, or see to their own wants; imbecility being applied to cases of weakmindedness more akin to those described as backward children. Generally speaking the term imbecility is applied to those who can speak, but where the power of speech is not attained the term idiocy is used. Much can be done to the very low grade of cases by proper nursing and a well-considered system of education specially adapted to their needs. When brought young under the influence of proper training some may be educated so as to be partly self-supporting, and the discipline and treatment applied to these cases may be seen in actual practice at such an establishment as the well-reputed Earlswood Asylum, not far from London, a visit to which conveys far more information than can be imparted within the scope of a lecture.

The personal duties of the nurse to individual patients, include her duties in regard to (1) delusions, (2) struggles, (3) precautions as to suicide, and (4) habits of insane patients.

As to (1) *delusions*, the nurse has a special *rôle*, for she should never ridicule a patient's delusions, however much harmless amusement may be derived from listening to them. Delusions result from morbid brain action. They are the consequence of disease and an indication of impaired mental action, and should not be laughed at. It is wiser to ignore them than to argue about or to contradict them. An effort should be made to turn the conversation into other directions and thus prevent the false ideas from being constantly before the mind. In this way a patient often ceases to refer to her insane ideas and eventually loses them. On no account should "nick-names," founded upon insane ideas, be given to patients, although they may refuse to reply to their proper names when under the influence of delusions of double personality, such as frequently occurs with delusions of identity. When morbid ideas cause patients to dress fantastically, personal efforts should be made to check them, otherwise these ideas become more fixed and may perhaps cause them to become destructive and tear clothing and other property wherewith to ornament their persons in accordance with their delusions. Such destruction and self-ornamentation, if unchecked, tend to encourage their morbid fancies, and to emphasise the differences between them and other patients which often cause satirical remarks to be made of an irritating or exasperating character. Here one may be pardoned for referring to "teasing," which is a temptation that every nurse should sternly resist. Not infrequently the humour of the situation may prove too much for young and inexperienced nurses, who tend to look upon patients only as eccentric persons whom they may unmeaningly banter. Such a weakness is unworthy of the mental nurse, and is discreditable when applied to the insane. The experienced nurse is never guilty of such a breach of trust and she discountenances this in others. It must always be remembered that those who have become inmates of lunatic asylums have already suffered sufficiently for their delusions by incarceration, segregation, and compulsory withdrawal from all ties that may be near and dear to them.

As to the duties of the mental nurse in case of (2) *struggles*, especial care should be taken immediately to summon sufficient assistance, as a violent and impulsive patient is often over-awed by the presence of two or three nurses, and yields rather than opposes, which she would do if the nurse were alone. The insane recognise the moral influence of superior physical force quite as much as do the sane. Struggles are equally a danger to the nurse as well as to the patient, and they should always be avoided if this be possible; moreover, struggles create a bad feeling between patients and staff, and it is wisest and best, rather than run the risk of personal encounters, to leave a patient alone while morbidly irritable and quarrelsome, as so often happens with epileptics after a fit. If left alone the irritability may pass off without involving a feeling of submission which may leave a rankling humiliation in the patient's mind; for some deluded patients are very vindictive, and they harbour imaginary as well as real grievances for long periods of time.

The precautions in regard to (3) *suicide* form a very important part of the duty of the mental nurse. Knives, scissors and all forms of cutting instruments should be kept under lock and key. Strings, shoe-laces, and articles of neck-wear; pails, basins, and water-taps must be kept from the reach of those patients who are suspected of suicidal tendencies. The greatest caution should be exercised with reference to the custody of medicines, lotions, and disinfectants, which should always be specially safeguarded. The patients in asylums who are known to have suicidal propensities are usually the objects of special instructions by the doctors, and they are identified by means of special case-papers, called "caution cards" or "suicidal tickets," which bear the patients' names, and state how they are to be supervised and what form of suicide they are predisposed to. Such patients are never allowed to go out of the nurses' sight for any time and they are even followed into the lavatories, and when one nurse relinquishes charge to another the patient is definitely and formally handed over and duly acknowledged—often in writing—which indicates the strictness of the personal supervision required from the nurse. Care is always taken in bedding such patients. They are carefully searched before retiring and their clothing and pockets examined after they have gone to bed, and in the morning before they rise, in case anything which may prove hurtful to them has been secreted.

As to (4) *habits*: in certain cases of depression patients may habitually refuse food, although much may often be done by tact in persuading patients to take it. If food is persistently refused the doctor then feeds artificially. In cases where the habits are defective, from mental weakness rather than from bodily infirmity or paralysis, care must be personally taken to prevent bed-sores; much may be done by the nurse in training such patients to attend to the calls of nature at regular intervals, and this training not only ensures improved and more correct habits but also saves the nurse trouble and promotes the physical comfort of the patient as well as tending to improve the mental state, for improved habits may be the first indication of convalescence in mental cases, showing that the exertions of the nurse are being successfully applied.

### "View Day" at St. Bartholomew's Hospital.

BY A BART'S NURSE.

The annual inspection of St. Bartholomew's Hospital by the governors and almoners, accompanied by the treasurer, steward, matron, and all the visiting and resident staff, is a most quaint and interesting ceremony. It has been observed for many generations every year on the second Wednesday in May, and the lucky visitor to the old hospital of Saint Bartholomew on "View Day" not only spends a most delightful afternoon within its ancient portals, but also carries away very pleasant recollections of the cosy, bright, flower-decked wards, and of many happy faces amongst the suffering ones in the neat white beds. For days beforehand all the nurses are busy washing, scrubbing, and cleaning everything which can be washed, scrubbed, and cleaned, all spare minutes occupied in making new cushion covers and locker cloths, smart flannel jackets and pinafores for the "cottys," and heaps of other articles to make the wards bright and pretty. All the doctors, sister and nurses subscribe towards expenses in their own ward. "View Day" is St. Bartholomew's gala day and no nurse will willingly absent herself from the hospital, however great the temptation.

Half the nurses and many of the sisters are up and off to Covent Garden Market before 5 A.M., bent on catching the early worm—in other words, taking the flower vendors by storm in quest of bargains. Everyone is in gay good humour, trying to keep their special style of ward decoration a secret from the others, and much fun is caused on coming across a sister surreptitiously buying up a barrow-load of blue forget-me-nots and iris. "Ah, sister, now we know your secret," and sister laughingly replies, "Too bad of you all to play Paul Pry." By a quarter past six everyone has to think of returning to hospital. After safely depositing their precious burdens in the wards, the nurses go to breakfast and are on duty as usual at 7 o'clock. The morning's work is got through as quickly as possible, and then comes the pleasant task of arranging the flowers, the pots and vases having been washed overnight. A small table is arranged in the centre of the ward with pens, ink, and blotting paper for the treasurer, and by half-past one everything is finished. Tiny Molly (who has been run over) has quite forgotten the pain in her poor little crushed legs, and is serenely stroking her "pitty noo pinny," whilst five-year-old Bobby is beaming in proud possession of "a ankercher" (for the first time in his life) just discovered in the pocket of his new blouse. Sister and nurses are standing ready and waiting to receive the committee, who arrive in a solemn and imposing procession, headed by the chief beadle in long, black gown, and bearing the long, silver-topped mace or staff of office. After calling out the name of the ward he retires to the background.

Next in order come the matron and treasurer, the steward of the hospital, the physicians or surgeons in charge of the ward, and behind those all the Governors and almoners and the students and dressers belonging to the ward. The treasurer then reads out the names of all patients at present in the ward, from a list handed to him by the steward. As each name is called out, the physician or surgeon in charge of the case states what the patient is suffering from and the probable length of his stay in the hospital. After this the treasurer asks the physican or surgeon if he is satisfied with the medical and nursing staff and their care of his patients during the past year. The matron is then asked if she has any fault to find with the nursing staff. The sister is also asked the same question, and finally the patients are requested by the steward "If they have anything to say or any fault to find to place it now before the committee."

It is on record that a little pneumonia patient once called out, "Please can I get up now and have meat and taters for dinner like No. 15?" There is a prize of £5 and two smaller sums, known as the Bentley prize, given on this day every year to the three nurses who have been longest in the service of the hospital, the treasurer himself congratulating the recipients.

After the committee have gone the ward is open to receive visitors. These are chiefly the nurses' and doctors' friends who are there by special invitation, but any visitor to the hospital on "view day" is most cordially welcomed and entertained. The patients, however, receive their visitors on Thursday instead of Wednesday—their usual visiting day. A dainty tea is served to the visitors in all the wards—the ward kitchen being transformed for the occasion. The patients also have high tea—those who are well enough—to make up for being kept in bed all day. The nurses now take it in turns to show their friends over the hospital.

The wards on "View Day" present a series of charming pictures, flowers being the only decoration used. One ward has great branches and sprays of apple blossom and pink hawthorn on mantel-shelf and window-ledges. Pale pink roses and carnations with trails of smilax are reflected in the glass-topped tables and dressing-wagons. Add to this the spotless white beds, with patients in gay bed-jackets and buttonholes of pink blossoms, a bright fire burning cheerfully in the large green-tiled stove, easy chairs with plenty of cosy cushions, and every brass knob and fixture polished like gold, the cool green walls for background, with some half-dozen well-chosen engravings—and you have a picture which it is almost impossible to believe exists in the heart of the City of London. Another ward is a mass of blue forget-me-nots and wild hyacinths, with clusters of blue clematis twined round the patients' pulley ropes. Another chooses white and gold, tall white lilies and narcissi, yellow marguerites and buttercups. In this ward each patient has a little nosegay of his own on the locker by his bed. The operating theatres deserve a peep. Everything spick and span and arranged as though just ready to receive a patient.

The dispensary is also well worth a visit. There one can see how the various drugs, pills, and medicines are prepared, the machinery being in full working order and the dispensers kindly explaining everything. The visitor may sample various aerated drinks made on the premises for the patients in the wards. No one should leave the hospital without visiting the Grand Hall, which contains a whole gallery of valuable portraits, including one of King Henry VIII. by Holbein. The hall is oak panelled and lofty, and holds several hundred people. All the banquets, entertainments, and some of the examinations are held here. Close by, just within the Smithfield Gate, is the quaint little church of St. Bartholomew the Less (a small parish of its own). Part of the tower still standing was built in the twelfth century. The church is open for prayer at all times.

### Asylum Workers' Association.

### ANNUAL MEETING.

There was a crowded attendance at the annual meeting of this Association on Tuesday, when Sir James Crichton-Browne took the chair as president for the last time. Among those present were Sir Robert Hensley, Chairman of the Metropolitan Asylums Board, Lady William Lennox, Dr Farquharson, M.P., Dr. and Mrs. Shuttleworth, Miss Honnor Morten, and many others.

In moving the adoption of the report Sir James Crichton-Browne said that there had been a decrease in the number of members, the figures being 3,691 as compared with 4,902 in 1902. This falling off was partly no doubt due to the small addition that had been made to the subscription, but this he considered very short-sighted of the members. The existence of the Association raised the status and intensified the *esprit de corps* of asylum workers; it promoted a feeling of fellowship among them, and when it was properly supported, when it numbered 7,000 or 8,000, it would, while still keeping strictly within its own province become a powerful agency for defending the just rights and promoting the interests of its members. A signal proof of the influence the Association was already exerting was given in the fact that in both the bills then before Parliament for the State Registration of Nurses, provision was made for a representation of the Association on the Central Board which was to be charged with the supervision of the education, discipline, and regulation generally of registered nurses throughout the country. He ventured to say that had this Association not been in existence, no such recognition would have been conceded to mental nurses. Neither of the bills before Parliament was likely to pass this session, but he believed that legislation on the subject could not be far distant, and that in that legislation the claims of asylum nurses would be properly recognised. No doubt there was strong opposition to the registration of nurses by hospital training schools, but in his opinion the same objections might be made to the registration of medical men or midwives. The plea that personal characteristics should not be overlooked he met by saying that all the most perfect personal characteristics possible could not make up for the lack of special training. The Medico-Psychological Society had already started registration and a general scheme of examination. Through the influence of the Association they were assured that when the next Lunacy Acts Amendment Bill was passed the scheme of assured pensions would be included. It was his conviction, after an experience of many years, that mental and nervous diseases were on the increase throughout the country. The reforms that were necessary in the lunacy laws had reference to the sweeping away of unnecessary restrictions that hampered the treatment of the patient at the present time. Whatever improvements were made in the status and well-being of the asylum workers reflected back favourably on the patients. He refused to admit that the introduction of women nurses into male wards only took place in 1896. He himself remembered to have reported on them thirty years ago. He alluded with gratification to the fact that twenty of their nurses were present at a conversazione of the Royal British Nurses' Association, when they had the honour of being presented to Princess Christian. This was, he believed, the first time that asylum workers had come into contact with Royalty since George I. paid a visit to Hanwell. He drew attention to the Reading Union and wished more members would join it. Twenty-one nurses and attendants had benefited by the Rest Fund, and many more should do so were the funds larger. In conclusion, Sir James observed that it was his last appearance in the chair, which he had occupied for seven years with great pride and satisfaction. He resigned it because he felt that others should enjoy the honour attached to that position, and also because he wished them to have some new blood in the Association. But he had not and never would resign his interest in its work and hoped yet to be able to serve it in a private position.

Loud and continued applause broke out when Sir James sat down.

The adoption of the report having been seconded by Dr. Sydney Coulard, Dr. Farquharson, M.P., moved a vote of thanks to Sir James Crichton-Browne for the admirable manner in which he had performed his work as president. This was seconded by Sir Robert Hensley, who said that he never went through an asylum without feeling sorry that he could not take the public with him that they might see how admirably things were conducted in asylums. It had been alleged that the nurses and attendants only did their work for the sake of money, but he denied this, and said the staff were animated by the highest feelings of duty. The marked improvement in the attitude of asylum workers must be attributed to the impulse of the Association.

Sir John Batty Tuke, M.P., was then elected as President for the ensuing year.

Other business having been despatched, the gold and silver medals were distributed to four attendants and nurses, as mentioned in our last issue. A vote of thanks to the Chairman closed the proceedings, and tea was then served to the company by Miss Shuttleworth and Miss Morten.

### Examination Questions for Nurses in the Colonies and Abroad Generally.

THE question was as follows:—"State what curative treatment you should pursue in cases of slight and also of serious bed-sores."

FIRST PRIZE.

Bed-sores are best treated by great cleanliness both of patient and bed-linen. Wash the skin exposed to discharges with warm boric lotion two or three times a day, rubbing in rectified spirits, brandy, or eau-de-cologne, and dusting with oxide of zinc and starch in equal parts, boric acid, or violet powder.

The pressure of the skin over the sacrum or trochanters is prevented by placing a ring of soft felt, covered on one side with adhesive plaster, round the prominent bone. The pressure of the body should be evenly distributed over its under surface by placing the patient on a water-bed or an air-bed at the commencement. Failing this a water-cushion, or ring-shaped air-cushion, should be placed under the pelvis, and by this means undue pressure at one spot will be prevented. The patient's position in bed must be altered frequently, and great care be taken in giving the bed-pan, which should be covered with a piece of soft flannel.

If, however, the skin breaks in spite of every precaution, oxide of zinc should be dusted on the sore part, covered with a piece of lint cut the exact shape and size of the wound, a thin layer of cotton-wool placed over it, and painted over with collodion. If there is much inflammation, boric ointment should be used instead of oxide of zinc.

If the skin sloughs and a large sore is formed, wash with a weak solution of mercury (1 in 7,000) three times a day and dress with zinc ointment, fixing on the dressings with adhesive plaster. The patient should, if possible, be made to lie on his side with a pillow to support the back and keep him from rolling over again. In cases which must be kept continuously in one position, a ring-shaped air-cushion or circular pad should be placed so that the sore rests in the centre of the circle.

In cases of ankylostomiasis (which is common among the Tamil coolies) it is very hard to prevent or cure bed-sores; for, these patients being usually dirty in their habits during health, are more liable than others to get bedsores. Being in such a weak, debilitated condition, the slightest irritation makes the skin slough away in large portions. Spirits and powder seem to have no effect on their tough skins. They object to lying on even a hard wooden bed and beg to be carried on to the floor of the outer verandah, where they seem perfectly happy lying in the sunshine.

All that can be done for these patients is to sponge them thoroughly twice a day with Condy's fluid, wash the sores with equal parts of creoline and mercury lotion (creoline ʒj to Oj of water and Hydr. per 1 in 4,000) and dress with zinc ointment.

Care should be taken to change the position of the patient frequently, for, the œdema being all over the body, even the knuckles, wrists, knees, etc., are likely to get sore. With care and good, nourishing food life may be prolonged, but the sores are seldom thoroughly healed.

"A PROBATIONER."

SECOND PRIZE.

Place the patient on a water-pillow, and use a ring-pillow with a hole, to prevent pressure on the affected part.

The sore should be carefully bathed in an antiseptic lotion, creoline or boric acid preferred; boracic ointment spread on the smooth side of lint, cut the size of the wound, should be applied, covered with a slightly larger piece of lint (dry) and strapped on with strips of adhesive plaster. The night garment should be turned up at the back to avoid lying on folds, and the draw-sheet kept smooth and free from crumbs, if sore is on back or hips. The dressing should be done once a day, unless there is incontinence, and then as often as it gets soaked.

In the treatment of a "serious bed-sore," should it occur on the back or hips, cut the night garment short, up to the lumbar region, to avoid wrinkles, and keep the draw-sheet smooth and free from crumbs. The patient should lie on a water-pillow, with a ring-pillow placed in position so that the sore and surrounding parts be free from pressure. In case of a slough, apply a poultice of crushed linseed and powdered charcoal, mix one part charcoal to six of linseed (dry), and make poultice fairly moist, apply, and cover with jaconet and layer of cotton wool, taking care to exclude the air, then put on a T bandage. In a deep wound, pack with cyanide gauze and cover with boracic ointment spread on lint, then strap with strips of adhesive plaster. In both cases irrigate with an antiseptic lotion. This treatment would apply to bed-sores on the heels or elbows.

Poultices should be changed every four hours, and dry dressing twice daily, more frequently in cases of incontinence.

"NEW ZEALANDER."

DEFECTS AND MERITS OF THE PAPERS.

"A Probationer" gains the first prize, as furnishing the best all round paper, though she, in common with almost every competitor from beyond the seas, advocates collodion and strapping.

"New Zealander" gains the second prize. She also is far too fond of plaster, but says nothing of collodion, which is an improvement.

HONOURABLE MENTION.

This is gained by "Z. Z. Z." who had the same distinction last year, and "Hey What." The latter sends a very good paper, but unfortunately she did not read the question carefully, and so has directed her answer largely to the "prevention" instead of the "cure" of bed-sores.

QUESTION FOR OCTOBER.

If called upon to nurse a case of scarlet fever in a house where there were other inmates, what steps should you take for the prevention of the spread of infection?

THE EXAMINER.

RULES.

The competition is open to all. Answers must not exceed 500 words, and must be written on one side of the paper only. The pseudonym, as well as the proper name and address, must be written on the *same* paper, and *not on a separate sheet.* Papers may be sent in for fifteen days only from the day of the publication of the question. All illustrations strictly prohibited. Failure to comply with these rules will disqualify the candidate for competition. Prizes will be awarded for the best two answers. Papers to be sent to "The Editor," with "Examination" written on the left-hand corner of the envelope.

In addition to two prizes honourable mention cards will be awarded to those who have sent in exceptionally good papers.

Six months is allowed in which to send in papers, but all answers must reach the office not later than September 20th.

N.B.—The decision of the examiner is final, and no correspondence on the subject can be entertained.

NEW RULE.

Any competitor having gained three prizes within the current year shall be disqualified from taking another until 12 months shall have expired since the first prize was gained.

### Hospital Requisites.

ANDERSON, ANDERSON, AND ANDERSON, LTD., have just opened West End premises at 58 and 59 Charing Cross, where their water beds, sheetings, and other hospital requisites can be obtained.

### Everybody's Opinion.

[Correspondence on all subjects is invited, but we cannot in any way be responsible for the opinions expressed by our correspondents. No communication can be entertained if the name and address of the correspondent are not given as a guarantee of good faith, but not necessarily for publication. All correspondents should write on one side of the paper only.]

### A QUESTION FOR THE METROPOLITAN ASYLUMS BOARD.

"Another Trained Nurse" writes: Allow me to modify the statement of a trained nurse; there is at least one hospital under the Metropolitan Asylums Board where the nurses' clothing is disinfected when they leave and where the matron satisfies herself on that point. And although the indoor and outdoor clothing is kept in the same room, the uniform is not allowed in the cupboards or drawers where the outdoor clothing is kept.

### TRAINING IN MATERNITY WORK AND CERTIFICATES.

The organising secretary of the Maternity Charity and District Nurses' Home, Plaistow, writes: "In a reply in 'Notes and Queries' given last week I notice you state that we grant a certificate for maternity and district work at the end of short periods of training. As a matter of fact we do not grant certificates for district nursing except to nurses sent by and working under associations and other public bodies, and then only at the end of three years' work. No certificate for midwifery is given, and the object of the monthly nursing certificate is to certify that the student has received instructions in certain subjects for a certain time."

### THE COLONIAL NURSING ASSOCIATION.

"L. M." writes: Acting upon your advice in The Hospital on Nursing in South Africa, I went to the Colonial Nursing Office at the Imperial Institute, and was told that as I had made no appointment I could not be attended to. Mentioning South Africa, and asking for pamphlets on the subject, I was referred to the office for the British Women's Emigration Association, and was there told that they could give me no information on the subject, but that I must go to 47 Victoria Street, S.W. As my time off duty had expired, I did not go to Victoria Street, and was called to Eastbourne next day, but seeing the same advice given again I felt that I must write and tell you, as a nurse has too little off-duty time to waste on such fruitless errands. I may add that at the office of the Colonial Nursing Association the secretary would have bidden me come again next day without even inquiring what my business was if I had not volunteered the information.

[When we advise correspondents to "apply" to an office, we mean, of course, that a written application for an appointment should be made.—Ed. Hospital.]

### STATE REGISTRATION.

Miss Amy Hughes writes: So much is being said and written on the question of State Registration that busy nurses find they have not time to unravel the tangled skein of conflicting opinions and judge for themselves how this movement will affect each one of them personally. Every nurse should be able to answer three questions. 1. What is State Registration? 2. How will it affect me? 3. What are the present difficulties? 1. "State Registration" means obtaining by Act of Parliament protection by law for those who are qualified to call themselves "registered nurses." The two Bills now before Parliament define "registered nurses" as those who can produce evidence of satisfactory training and pass an examination instituted by a Central Board or Council. One Bill asks for three years' training in hospital wards, the other for three years' training evidenced by certificates. Both Bills ask (*a*) That anyone pretending to be a registered nurse and acting as such may be punished by fine or imprisonment. (*b*) That the Central Board or Council may be given power to suspend a nurse or remove her name from the register for disobedience to the rules or other misconduct. 2. How will State Registration affect nurses now at work? Nothing in either Bill affects those whose nursing knowledge has been gained outside hospital or infirmary wards. These nurses are not eligible for the register, and so long as they do not claim to be "registered nurses" are free to work as they will. A large body of nurses do not hold certificates of training from a hospital or infirmary training school. Some, for good and sufficient reasons, were unable to complete the full term of training. Some passed from institution to institution as "paying probationer" or otherwise. Some received hospital training for a time in connection with private nursing institutions, completing their agreement as private nurses. In addition to nurses holding the full certificates of recognised training schools, there are many with certificates from minor hospitals, some from cottage hospitals with few beds, and many with certificates from the smaller Poor-law infirmaries. For all of these grades of training provision is made in both Bills. All nurses at work when the Act is passed will be given a "time of grace" in which period of two years every nurse can decide what she will do about registering herself. There will be no examination for nurses then at work, but a fee for registration will be made. One Bill states that the qualifications for these nurses should be: Three years' certificate from an approved hospital and good character, or evidence of satisfactory training, three years' in bonâ fide practice as a nurse and good character. The other Bill states: Two years' recognised certificate, or satisfactoy evidence of bonâ-fide practice as a trained nurse and good character. Therefore under neither Bill would any hardship be inflicted on the present nurses and those working when the Act became law. With very few exceptions all can meet one or other of the proposed conditions of registration. 3. The present difficulties are:—1. The strong reasons urged for and against the principle of State registration. 2. The methods proposed by the present Bills. (1) The reasons urged in favour of registration are:—(*a*) The desirability of a central body to decide the minimum course of training for nurses, and to maintain a professional standard and common rules of discipline in the nursing profession. (*b*) The protection of the public from fraudulent and unscrupulous exploiters of half-trained and untrained persons trading as fully qualified nurses. (*c*) The protection of nurses who have given time and money to learn their profession thoroughly and find themselves competing for a livelihood in the same market for the same remuneration as those who have spent a few months in the work. (*d*) An examination in addition to certificates of training would satisfy the public and medical men that nurses on the Register possess the required practical and technical knowledge to carry out all instructions skilfully and intelligently. Such an entrance examination would lead to nurses in smaller hospitals being prepared to pass it equally with those in the large training schools, and thus raise the standard of nursing generally. The reasons urged against registration are:—(*a*) That a Central Board is unnecessary, as the large hospital training schools are quite sufficient in themselves to keep up the nursing standard. (*b*) That the proposed register would be dangerous to, instead of protecting, the public, as it would be difficult, if not impossible, to remove an undesirable nurse, and once registered, she would be accepted without further inquiry. (*c*) That an examination for admittance on the register would keep out many good nurses, and give the first chance to clever but otherwise undesirable persons, as examinations cannot test the many moral qualities necessary for a good nurse. 2. The proposed construction of the Central Board is a difficulty to numbers who wish for State registration, but do not approve of the present Bills. Few or no lay members are suggested, which is not approved by many. Another objection is fixing the present standard of training too definitely. The conditions should be such that the nursing standard is left free to develop as required to meet all future demands of medical science. In this outlining State registration, its effects and difficulties, I would urge all nurses, but especially the large body of private nurses which is directly in touch with the general public, to think over this great movement not only with regard to themselves, but to the nursing world as a whole. The feeling that has been aroused shows that the public are not satisfied with the nursing profession in its present condition. The time has come when those who demand and pay for a fully-qualified nurse should possess a guarantee that they obtain the person they require. The time has come when women who take up the responsible calling of a nurse should have a guarantee that the certificate which they give time and strength to earn shall receive its market value in the outside world. It is also time that those who for lack of room or opportunity are not trained in a large hospital may prove they possess the skill and knowledge which render them competent for their work. No amount of training, examination nor registration can turn an undesirable into a desirable nurse, but neither do moral and womanly qualities enable their possessor to meet the heavy responsibilities of her position without the discipline and training which alone can fit her to undertake it with safety to those entrusted to her care. It is for us to decide for ourselves. Shall we endeavour by means of legislation to ensure we acquire thorough practical knowledge of our work, and submit it to the test of a common examination? Will the institution of a central body with disciplinary powers be for the advantage and protection of the general public and trained nurses alike? On our answers State Registration stands or falls.

MISS ANNIE CARTER, matron of Cottage Hospital, Bromley, Kent, writes: The question of State registration has been much discussed in your columns and many have written in opposition to it. I should like to say a few words in favour of it. I believe that State registration would be a great protection to the nursing profession, and to the public generally. So many, in writing against it, have said that the personal qualities of a nurse will not be considered in registration. I do not see how that would be possible. Surely the matron under whom the nurse has trained should be the best judge of her in that respect; if the nurse is personally unfit to be registered as a good, trustworthy nurse I do not consider it just on the part of the hospital authorities to have kept her for the three or four years of her training and to have given her a satisfactory certificate; after all, no woman can be conscientiously certified as to thoroughly good nurse without consideration being given a her personal qualities—in fact, I consider they play a very important part in the making of a valuable nurse. Surely State registration must be a great boon to the public, as it will no longer then be possible, when applying to a nursing institution for a trained nurse, to be put off with a nurse who has had as little as one year's training, and that perhaps is a very small hospital, or, worse still, in a fever hospital. These partly-trained women are sent to any cases, however important, and take their places in every way on a level with women who have undergone a training of three or four years. This I consider brings into great disrepute—first, the institution by whom they are employed and sent out; secondly, the whole of the nursing profession. State registration will guarantee a good, well-trained, efficient nurse; surely that should be a comfort to patient and friends, even if her personal qualities are not exactly what they would wish. Professional capabilities are of the greatest importance when nursing a critical case.

### A WARNING TO MATRONS OF PRIVATE NURSING INSTITUTIONS.

"ANOTHER NURSE" writes: In corroboration of an "Indignant Nurse's" remarks respecting a so-called nurses' and nursing institution in Lancashire, I myself had the extreme misfortune to join what I imagine by "Indignant Nurse's" statement to be the same institution in Lancashire, and can truly endorse her statement respecting the food which was most inferior both in quality and quantity, and all things in general most comfortless. I agree with "Indignant Nurse" that there are always two sides to a question, and I feel that we, as nurses, are justified in publishing our complaints when occasion arises.

### FILLING A WATER-BED.

"INFIRMARY NURSE" writes: I should like to hear the opinions of nurses as to my method of filling a water-bed. I place the patient (feet to the head) on the bed next the one I am preparing for him. The mattress, which is an ordinary one, I cover with a good mackintosh. I place the water-bed on this, and fill it with warm water from a pail by the bedside. I then cover the whole with a sheet and draw-sheet, and place the patient in bed with very little, if any, difficulty.

### SILENCE IS GOLDEN.

"SISTER" writes: Nurses when off duty often do their own profession great harm by their thoughtless, and alas! frequently untruthful, conversations. Opinions are loudly passed, perhaps on the top of a tram, on a local doctor and how he has, in their superior knowledge, bungled such and such a case. Very likely, were they cross-examined on the said statements, there would be found utter ignorance of the why and wherefore of the doctor's treatment. Nurses are far too prone to give opinions, and pass comments to patients and friends, of doctors' treatments, without first thoroughly knowing why they do so. When do we hear doctors discussing nurses' failures and woful mistakes—mistakes almost incredible, to believe possible, such as burning a patient with hot-water bottle, etc., etc. Doctors are never too old to learn, neither, I hope, are nurses. A wise, and kind, truthful tongue is a prize in any nurse. Nurses do not add to their attractiveness nor show off their cleverness by talking of treatment prescribed by doctors.

### PRINCESS MARY VILLAGE HOMES.

"ANOTHER CHARGE NURSE" writes: In answer to "Charge Nurse," I think if she were to put herself in the place of the assistant superintendent, she would feel that that lady had asked no more than her right. When she is acting for the superintendent she is entitled to the same respect and obedience, even if she is not a trained nurse. If in case of illness she is not to be informed, how is she to know what is going on in the homes? Of course, when the superintendent is on duty, the nurse should be answerable to her only.

### GOING UP FOR THE L.O.S.

"NURSE M." writes: After having to obtain two references as to my moral character, and a doctor's certificate for examining over twenty cases, and answering heaps of other questions, I was accepted as a candidate for the London Obstetrical Society, and informed there were two examinations, written and oral. I had studied hard to obtain the much-coveted L.O.S. certificate, but as the time drew near to go up for my first examination, I began to get very nervous and very much dreaded the ordeal. So it was with trembling steps I entered 20 Hanover Square. A kind and gentle-faced lady met me—and my fellow candidates—in the hall. We were then marched two abreast into a large room, full of long tables, and each given a paper with five or six questions on it. We were next requested not to speak a single word to each other; the room was so quiet one could have heard a pin drop. We were allowed, as far as I can remember, two hours to answer the questions. My spirits dropped to zero when I read them over, but I set to work with a will to answer them to the best of my ability, and was surprised to find that I was the first to finish. I got out as quickly as possible, just in time to catch the last train for the Midlands. A week or so later I returned for the oral examination, which, to my mind, is far more difficult. We were ushered this time into a large room at the bottom of the hall to await our turn to be admitted into the examination room. My feelings were not cheered when I saw several nurses return looking pale and miserable and a whisper of failure reached my ears as they passed out. After what seemed to be hours of suspense my name was called, and with four or five other nurses I was escorted to the examination room by the same kindly faced lady, we were each placed at separate small tables where very solemn-looking doctors awaited us. A pelvis and fœtal skull were on each table, but I forgot these and the occupants of the room in my own anxiety. After being questioned severely on transverse presentations, placenta prævia, breech cases, and heaps of other things, which seemed to last an endless time but in reality did not occupy more than 15 or 20 minutes, the doctor wrote on a slip of paper and handed it me and sent me back to the large room when I was informed I had passed. I was so excited that I could scarcely write my name and address when requested. Two or three of us had quite a race through the street to find the nearest post office to wire to our friends to inform them of our success.

### Appointments.

[No charge is made for announcements under this head, and we are always glad to receive, and publish, appointments. The information, to insure accuracy, should be sent from the nurses themselves, and we cannot undertake to correct official announcements which may happen to be inaccurate. It is essential that in all cases the school of training should be given.]

Ancoats Hospital Convalescent Home, Sandlebridge, Lancashire.—Miss Florence M. Smith has been appointed nurse-matron. She was trained at the Western Infirmary, Glasgow, and the Fever Hospital, Glasgow. She has been nurse-matron of the Cottage Hospital, Keswick, and latterly of the Cottage Hospital, Hayes, Middlesex.

Brighton, Hove, and Sussex Throat and Ear Hospital.—Miss Gertrude A. Hemrick has been appointed matron. She was trained at St. Thomas's Hospital, London, and has since held several staff appointments in the male and female medical and surgical wards.

Coventry City Isolation Hospitals.—Miss Jessie E. Ashe and Miss Isabella Fardon have been appointed sisters. Miss Ashe was trained at the Chorlton Union Hospital, Manchester, and has since been charge nurse at Prescot Infirmary, charge nurse at one of the hospitals under the Metropolitan Asylums Board, and sister at the City of Glasgow Fever Hospital, Ruchill. She holds the L.O.S. certificate. Miss Fardon was trained at the Warneford Hospital, Leamington, and was afterwards on the private nursing staff.

Isolation Hospital of the Orsett Joint Board.—Mrs. E. W. Taylor has been appointed nurse matron. She has been nurse at one of the hospitals under the Metropolitan Asylums Board.

Rothwell, Methley, and Hunslet Joint Hospital.—Miss Florence Weddall has been appointed matron. She was trained at St. George's Infirmary, Fulham Road, London. She has since been charge nurse at the Royal Hospital for Sick Children and Women, Bristol; night superintendent and deputy-matron at the City Isolation Hospital, Nottingham, and sister-in-charge of the City Small-Pox Hospital, Nottingham.

Southwark Union, London.—Miss Rosa Templeman has been appointed superintendent nurse. She was trained at Wandsworth and Clapham Union Infirmary, has been charge nurse at Cardiff Union Workhouse, charge nurse at Park Fever Hospital, London, head midwife at Lambeth Workhouse, and superintendent nurse at Braintree Infirmary.

Victoria Hospital, Accrington.—Miss Mary Carpenter has been appointed matron. She was trained at the Victoria Hospital, Burnley, where she has since been ward sister.

Weston-super-Mare Infectious Diseases Hospital.—Miss H. J. Mooney has been appointed matron. She was trained at Swansea General and Eye Hospital, where she was afterwards sister, night sister, and assistant to matron. She has since done fever and small-pox nursing, and has been nurse-matron at Rushcliff Isolation Hospital, Hucknall, Notts. She is at present engaged at Joyce Fever Hospital, Dartford.

### TRAVEL NOTES AND QUERIES.

By Our Travel Correspondent.

Yverdon in Switzerland (G. G. and H.).—Yverdon is reached *viâ* Neuchâtel. Its waters are considered good for affections of the pulmonary and vesical mucous membranes, but I cannot advise you. You must consult your medical man, it is most dangerous to choose health resorts of that kind for yourself. No, it is not at all expensive; accommodation can be had at the Hôtel de Londres for 6½ francs per day, also at the Hôtel Faucon and Hôtel Paon. There are two pensions still cheaper—Pension la Prairie and Pension Maison Blanche.

Switzerland and the Rhine (Natt).—I do not think there is any party starting in July which will give you what you want. Send a stamped and addressed envelope to Messrs. Cook and Sons, Ludgate Circus, and ask them if they have any suitable arrangement projected about that date. Almost all personally-conducted tours are for much shorter periods than a month. If you mean £20 between two of you, it cannot be managed. I conclude you mean £20 each. That is enough with careful management for a month's holiday on the continent, but I think you are making a mistake in trying to see Switzerland and the Rhine; so much travelling and so many changes of hotels make it expensive. Write to me more fully telling me your tastes, and I will arrange a tour for you.

Bude at the End of May (Sister H.).—This is a mildly pretty place, but has not the splendid coast scenery of Tintagel nor the artistic charm of Boscastle; it is, in fact, rather tame, but comfortable and fairly bracing. There are several hotels, and one boarding house called the "Erdiston." Go there or to the Falcon or Globe Inns and look for lodgings. I rather think the chief ones have a sea view, but are on the summit of the cliff, which might be a disadvantage. There are plently of excursions by coach and rail: 18 miles distant is Boscastle, reached by coach. This latter place is, I think, rather better placed for excursions; for instance, Tintagel could be easily visited in a day and many other interesting spots. Tell me if I can help further.

Tour in Devon or Cornwall (Nurses A. and B.).—I think Lynmouth or Lynton, and a few days at Clovelly, is what you would like. Lynmouth and Lynton are in the centre of very beautiful scenery, and the coach runs are not so expensive as you think. You reach these two places by coach from Barnstaple. Second-class return ticket from Waterloo to Barnstaple, £1 14s. 6d., rather less in the tourist season. Hotel at Lynmouth—Lyn Valley, private hotel; at Lynton, the Crown or Queen's, write for terms. Lynton is more bracing than Lynmouth; the latter more picturesque. The two are connected by a cliff railway. A week at Clovelly would be pleasant. It is reached *viâ* Barnstaple and Bideford, thence by coach. Try for accommodation at The Red Lion. Unfortunately you are taking your holiday at a very expensive time. Do not take your cycles, it would be more trouble than pleasure. If you prefer Dartmoor, write me again, and I will give you addresses. The amount you have at your disposal is rather small, and you do not tell me for what period it is to last. If these hotels are too dear, write to the Traffic Manager, at Waterloo, and ask for the South Western "Gazette of Farmhouses and Lodgings."

### To Nurses.

We invite contributions from any of our readers, and shall be glad to pay for "Notes on News from the Nursing World," or for articles describing nursing experiences at home or abroad dealing with any nursing question from an original point of view according to length. The minimum payment is 5s. Contributions on topical subjects are specially welcome. Notices of appointments, letters, entertainments, presentations, and deaths are not paid for, but we are always glad to receive them. All rejected manuscripts are returned in due course, and all payments for manuscripts used are made as early as possible after the beginning of each quarter.

### Echoes from the Outside World.

### Movements of Royalty.

THE King was represented at the funeral of Sir H. M. Stanley, on Tuesday, by the Hon. Sidney Greville, his Majesty's private secretary. Two letters have been written by his Majesty to Lady Stanley, in one of which he observes, "In Sir Henry Stanley the British Empire has lost an eminent Englishman and one who has performed great services, not only to his own country, but to the world, inasmuch as few men have done more to advance the cause of civilisation than Sir Henry. The King feels certain that Englishmen will always cherish Sir Henry's memory with affectionate regard." In the message of condolence sent by Queen Alexandra to Lady Stanley, her Majesty paid a high tribute to the services of the late explorer, and referred to the deceased as "a man of whom all England was justly proud, and whose name would be a household word in British homes for ever." On Monday Lord and Lady Curzon arrived in London on their return from India, and Lord Knollys was at Charing Cross Station to welcome them on behalf of the King. He also conveyed a message from the King commanding them to proceed direct to Buckingham Palace. At the Palace Lord and Lady Curzon were very cordially received by the King and Queen, and remained upwards of an hour.

### Russia and Japan.

THE docks and piers at Dalny, a city which Russia wanted to be the great mercantile emporium of the Far East, were last week destroyed in order to increase the difficulty of a Japanese landing. With regard to the charge of firing on a train from Port Arthur flying the Red Cross flag, the Tokyo Foreign Office state that the train carried no special marks; that Russian soldiers who were passengers fired on a detachment of Japanese, and that it was not until the latter replied that the Red Cross flag was hoisted. On Thursday of last week the first properly-equipped hospital train passed through Mukden for Harbin with 252 wounded. Twelve freight and passenger cars were converted for the use of the Russian wounded. The men were tended by nurses and Sisters of Charity. Admiral Kataoka reports that the despatch-boat *Miyako* was lost while assisting in the operation of clearing the Russian mines in Kerr Bay. She struck a mine which had not been detected, and sank in 22 minutes. Two sailors were killed and six wounded. The rest were rescued. General Kuroki reports the occupation by a detachment of his army of Kuan-tien-cheng, and the Japanese have occupied Sin-yen. The Czar has begun a personal tour of inspection of the troops quartered in certain provincial towns, his object being, it is presumed, to stimulate patriotic feeling. A message from the Russian headquarters at Mukden states that the fighting line is steadily nearing that city, where the Viceroy's headquarters still remain.

### Ladies as Barristers.

LAST week the annual "Ladies' Night Debate," under the auspices of the Hardwicke Society, took place in the Middle Temple Hall. The Society's President, Mr. P. B. Morie, was in the chair, and the debate, which was on the question whether the admission of women to the Bar was or was not a "subject proper for the deliberation of Parliament," was opened by Mr. Atherley-Jones, K.C., M.P. He maintained that the judicial decisions in England and Scotland against the admission of women to practise at the Bar was not to be regarded as final, and after pointing to the fact of their admission to such practice in some parts of America, in Canada, and in Italy, he submitted that there was no valid reason why the same privilege should not be extended to women in our own country. Mr. Plowden, the well-known police magistrate, discussed the idea of the admission of women to the Bar as "mad and mischievous," and said that "it was conceived in the worst interest of those delightful creatures whom it was meant to benefit." Miss Parnhurst, a law student, accused Mr. Plowden of unwarrantable levity, and told him that he did not know what the modern woman was like. Mr. H. C. Richards opposed the affirmative motion "as a friend of the ladies," Mr. Brownlow supported it, and Lady Hamilton took the same view as the chairman. On a show of hands there were 32 votes for it and 32 votes against, and the chairman, in accordance with the rules of the Society, gave his casting vote against.

### Joachim's Diamond Jubilee.

THE sixtieth anniversary of the first appearance of Dr. Joseph Joachim in this country was an event of surpassing interest. It took place on Monday in Queen's Hall, and a concert was given consisting mainly—by the great musician's desire—of compositions from the pen of the three great masters, with whom he was personally acquainted, Mendelssohn, Schumann, and Brahms. To mark Dr. Joachim's place among German composers the overture to "Henry IV." was included, and under the composer's able direction it went splendidly. One item in the programme was left blank, with the exception of the words "Violin solo—Dr. Joachim." It was a charming surprise for the audience when, in responding to the address in which Mr. Balfour begged the acceptance by the great violinist of his picture by Sargent, Dr. Joachim announced that he would "try" to play the work in which he first appeared at the Philharmonic concert in 1844, Beethoven's great Concerto. The performance was a remarkable one, although later the performer begged the indulgence of the audience for any shortcomings in his playing, caused by the natural excitement of such an occasion. He was presented with a very large "floral tribute" by little Franz von Vecsey, the 11-year old violinist who is at present making such a sensation in London.

### Lady Golf Champion.

THE 12th annual tournament for the ladies' open golf championship was brought to a close at Troon on Friday last, when the semi-final and final rounds were played. The final, which was played in the afternoon between Miss Lottie Dod, of the Moreton Club, Liverpool, and Miss Hezlet, of the Royal Portrush Club, produced a splendid match, Miss Dod winning by a put on the home green, after the players had stood "all square" at the 17th hole. A crowd of some 5,000 spectators followed the final, and at one time was so unruly that the play was stopped for awhile. Miss Dod has been lady champion of lawn tennis, and is a hockey player of note. She has improved greatly in golf during the past twelve months, and her success in carrying off the championship is admittedly well earned.

### Earl's Court Exhibition.

IF the fine weather of early May continues throughout the summer, the Earl's Court Exhibition this year should be a big success. Italy especially lends itself to picturesque treatment, and the management have made the show as realistic as possible. "Venice by Night" is arranged in the Empress Theatre, so that it is equally available wet or fine. There is three-quarters of a mile of canals and thirty-eight gondolas, made in Venice specially to order and manned by tall and swarthy gondoleri from the "City of Islands," are ready to transport passengers past the Ducal Palace, St. Mark's Church, the Bridge of Sighs, the Rialto Bridge, and the Grand Piazzo. Amongst other attractions the Blue Grotto of Capri is also represented. Other features are a realistic ascent of a volcano, a fine panorama of the Duke of the Abruzzi's expedition "Farthest North," theatrical performances at "La Scala," and Sir Hiram Maxim's new flying roundabout.

### Notes and Queries.

FOR REGULATIONS SEE PAGE 12.

*Epileptic.*

(51) Can you tell me of any charitable institution where a man suffering from epileptic fits could be received? He is a married man, about 40, and a baker by trade. He is industrious and fond of gardening.—*Woodfield.*

Write to the National Society for the Employment of Epileptics, 12 Buckingham Street, Strand, W.C. The colony is at Chalfont St. Peters, Bucks.

*South Africa.*

(52) I am desirous of obtaining a post either in a hospital or private institution. Will you kindly tell me where to apply?—*Nurse H.*

Write to the Secretary the South African Colonisation Society, 47 Victoria Street, Westminster, S.W.

*L.O.S.*

(53) Is it possible to take the L.O.S. certificate after preparation by correspondence and by attending the specified number of cases under a local certificated midwife?—*E. W.*

Submit your query to the Secretary of the London Obstetrical Society, 20 Hanover Square, W.

*Midwifery Experience.*

(54) I am taking a course of lectures in obstetrical nursing with a view of becoming qualified. Will you kindly tell me where I could go to get practical experience?—*C. B.*

You do not give your full address. If in London, the Secretary, the Midwives Institution, 12 Buckingham Street, Strand, W.C., could arrange matters for you.

*Home Offered.*

(55) A friend wishes to know the best medium through which to advertise for the care of a child. She can give medical and other references.—*N. B. F.*

Place the matter in the hands of a good advertising firm.

*Paris.*

(56) Will you kindly give me the address of the British Hospital in Paris?—*L. A. W.*

The Hertford British Hospital, Rue de Villiers-Levallois-Perret, Paris.

*America.*

(57) Please inform me if I could train in America in hospital nursing after training for three years in an asylum here.—*Eugam.*

It should be possible, but the question can only be answered by the authorities of the school to which you apply for training.

*Cancer.*

(58) Can you tell me of a home where a woman of good parentage, aged 45, suffering from cancer in the left arm, could be received? Her friends could pay a small amount, but she is quite without means.—*Suffering.*

See if the case is suitable for the Cancer Hospital, Fulham Road, Brompton, S.W.; or for the Middlesex Hospital, Mortimer Street, W. Otherwise consult "Burdett's Hospitals and Charities," under "Chronic and Incurable."

*Stammering.*

(59) Will you kindly tell me if stammering would prevent my being trained as a nurse in hospital?—*J. H.*

You can only ascertain this by enquiry at the hospitals.

*Pension Fund for Nurses.*

(60) Will you kindly give me the address of the Royal National Pension Fund for Nurses?—*E. P.*

28 Finsbury Pavement, E.C.

**Important Nursing Textbooks.**

"Nursing Profession: How and Where to Train." 2s. net; 2s. 4d. post free.

"Nurses' Dictionary of Medical Terms and Nursing Treatment." By Honnor Morten. 2s. post free.

"Nursing." By Isabel Hampton. 7s. 6d. net; 7s. 10d. post free.

"Surgical Ward-Work and Nursing." By Dr. Alex. Miles. 3s. 6d. net; 3s. 10d. post free.

"Outlines of Insanity." By F. H. Walmsley, M.D. Popular Edition. 1s. 6d. post free.

"Notes on Pharmacy and Dispensing for Nurses." By C. J. S. Thomson. 1s. post free.

The above works are obtainable of all booksellers, or direct from The Scientific Press, Limited, 28 & 29 Southampton Street, Strand, London, W.C.

### For Reading to the Sick.

### WHITSUNTIDE.

COME, Thou Holy Spirit, come;
And from Thy celestial home
Shed a ray of light Divine;
Come, Thou Father of the poor,
Come, Thou source of all our store,
Come, within our bosoms shine:

Thou of Comforters the best,
Thou the soul's most welcome guest,
Sweet refreshment here below;
In our labour rest most sweet,
Grateful coolness in the heat,
Solace in the midst of woe.

Heal our wounds; our strength renew:
On our dryness pour Thy dew;
Wash the stains of guilt away:
Bend the stubborn heart and will;
Melt the frozen, warm the chill;
Guide the steps that go astray.

On the faithful, who adore
And confess Thee, evermore
In Thy sevenfold gifts descend;
Give them virtue's sure reward,
Give them Thy salvation, Lord,
Give them joys that never end.

*From the Latin.*

The Spirit's presence is not given us to make up for an absent Christ: it is rather to secure to the faithful an effective spiritual presence of the Christ, Who, though invisible, will not be less, but even more truly with them; with Whom they will have closer and more endearing intercourse by faith, than sight or touch, in this world, could carry on.

*Dr. Bright.*

If anything can give a calm mind, disperse our scruples and fears, soften our cares, invigorate our actions, and fill our very words and looks with the joy of the Holy Spirit, it is this simple childlike trust in God. . . . God never said, "Walk before thyself,—but He did say, "Walk before Me, and be thou perfect; and David says, "Mine eyes are ever looking unto the Lord, for He shall pluck my feet out of the net." The danger is at his feet, but his eyes are raised on high—looking rather to God's help than to his own peril. In the sight of God all will be plain, but in our own darkness we can see nothing.—*Fenelon.*

For that Holy Spirit of order, as He pours His influence into us, has a definite work for our energy to spend itself upon, amidst all the vast and complicated machinery of the world; and Love is the initial, the foundation motive, which is to start our force, our passions, our motives, our imagination, our intellect, our strength, into their proper groove amidst the great labyrinth-scheme of the Providential working of God. Plato in his "Republic" wishes that his physicians should have had themselves all diseases, that they may help others by experience. So we have to unbind our heart-sores and reopen our wounds to comfort and help others, by the "comfort wherewith we ourselves are comforted of God."—*Canon Newbolt.*

